# MGRN1 in development and disease: a unifying view of a versatile membrane-tethered E3 ubiquitin ligase

**DOI:** 10.1042/BST20250109

**Published:** 2026-05-06

**Authors:** Alyssa Riglos, Teresa M. Gunn, Jennifer H. Kong

**Affiliations:** 1 Department of Biochemistry, School of Medicine, University of Washington, Seattle, Washington, U.S.A.; 2 McLaughlin Research Institute and Weissman Hood Institute at Touro University, Great Falls, Montana, U.S.A.

**Keywords:** E3 ubiquitin ligase, GPCR trafficking, MGRN1, receptor regulation, substrate specificity, ubiquitination

## Abstract

Mahogunin Ring Finger 1 (MGRN1) is a multifunctional E3 ubiquitin ligase with broad biological significance and belongs to a small group of membrane-tethered E3s capable of regulating signaling receptors at the plasma membrane. Studies in mice first revealed its physiological importance, as loss of *Mgrn1* leads to a wide range of phenotypes, including abnormal pigmentation, congenital malformations, and neurodegeneration. Remarkably, MGRN1 localizes to multiple cellular compartments, including the plasma membrane, mitochondria, nucleus, and endo-lysosomal pathway. MGRN1 is also involved in several cellular processes, including receptor regulation, protein homeostasis, and mitochondrial maintenance. While studies have emphasized the importance of MGRN1, it has been difficult to define unifying principles governing its function. In the present review, we summarize and integrate published findings to develop a clearer picture of MGRN1’s roles, focusing on phenotypes observed in mouse models and the signaling pathways MGRN1 regulates. We propose shared mechanistic themes that reconcile the functional diversity of this unique E3 ligase, highlight gaps in the current literature, and identify areas for further investigation to better understand MGRN1’s role in disease and evaluate its potential relevance for targeted protein degradation strategies.

## Introduction

E3 ubiquitin ligases are central regulators of protein homeostasis, providing substrate specificity within the ubiquitin-proteasome system [1]. Mahogunin Ring Finger 1 (MGRN1) is a member of the RING family of E3 ligases, defined by a conserved C3HC4 RING domain that catalyzes ubiquitin transfer from an E2 ubiquitin conjugating enzyme onto lysine residues in substrate proteins [2]. Unlike most RING E3 ligases, MGRN1 is N-terminally myristoylated. This lipid modification anchors it to membranes and enables interactions with membrane-associated proteins, thus positioning it to regulate receptor abundance and modulate signaling activity [3–6]. The presence of *Mgrn1/MGRN1* orthologs in nearly all major eukaryotic lineages suggests that it is an evolutionarily ancient ligase that performs essential yet flexible functions across different cellular contexts [4,7,8].

**Figure 1:**
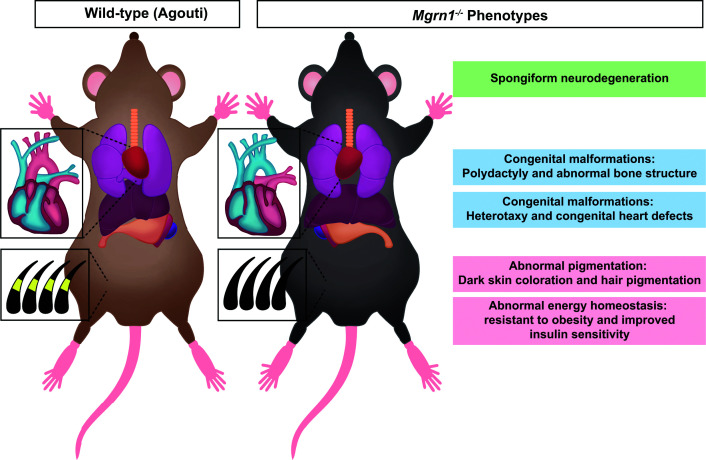
Overview of phenotypes associated with *Mgrn1^−/−^* mutant mice (Left) A schematic of a C3H/HeJ wild-type mouse with Agouti (brown) coat color and normal organ morphology. (Right) In contrast, the *Mgrn1^−/−^* mutant mouse has many phenotypes. These phenotypes underscore the essential contributions of MGRN1 to both embryonic patterning and adult tissue homeostasis.

The physiological significance of *Mgrn1* was first revealed when it was discovered as the gene mutated in the *mahoganoid* (*md*) mutant mouse, identified in the 1960s for its unusually dark coat color on a C3H/HeJ background that normally displays a brownish (Agouti) pattern [9] (Figure 1 and Table 1). Genetic interaction studies later showed that the *md* mutation could suppress both the pigmentary and metabolic phenotypes of the *lethal yellow* (*A^y^*) mutation, darkening coat color and reducing obesity in a gene dose-dependent manner [10,11]. These findings hinted at a broader regulatory role in melanocortin signaling, beyond melanocyte biology. Positional cloning ultimately identified *Mgrn1* as the gene disrupted in *md* mice [12], and subsequent characterization demonstrated that pigmentation and metabolic regulation represent only a fraction of MGRN1’s functions. Mice lacking *Mgrn1* exhibit a broad range of developmental and adult phenotypes, including congenital malformations and spongiform neurodegeneration [13,14] (Figure 1 and Table 1).

**Table 1:** Table of phenotypes in *Mgrn1^−/−^* mutant mice

Phenotype	Substrate	Pathway	Source
Abnormal cranium morphology	SMO	Hedgehog signaling	[31,56]
Abnormal bone structure	SMO	Hedgehog signaling	[4,56]
Abnormal ear morphology	SMO	Hedgehog signaling	[56]
Abnormal heart development	SMO	Hedgehog signaling	[4,13,56]
Preweaning lethal	SMO	Hedgehog signaling	[4,13]
Abnormal skin coloration	MC1R	Melanocortin signaling	[56,57]
Abnormal hair pigmentation	MC1R	Melanocortin signaling	[10,12,26,56,57]
Suppresses obesity and improves insulin sensitivity in yellow obese mouse strain (*A^y^*)	MC3R and MC4R	Melanocortin signaling	[11,58]
Spongiform neurodegeneration	TSG101	ESCRT-I function	[40]
Hypomyelination	TSG101	ESCRT-I function	[59]
Spongiform neurodegeneration	GP78	Mitochondrial function	[60]
Abnormal left-right patterning	Unknown	Nodal signaling	[4,13]
Increased mean corpuscular hemoglobin	Unknown	Unknown	IMPC^*^
Decreased circulating HDL cholesterol levels	Unknown	Unknown	IMPC
Abnormal auditory brainstem response	Unknown	Unknown	IMPC
Increased circulating bilirubin levels	Unknown	Unknown	IMPC
Increased circulating alkaline phosphatase levels	Unknown	Unknown	IMPC

* Phenotypes reported through the large-scale phenotyping efforts of the International Mouse Phenotyping Consortium (IMPC).

Despite the diverse phenotypes reported for *Mgrn1* mutant mice, the molecular logic by which MGRN1 exerts its effects remains challenging to reconcile. *Mgrn1* is broadly expressed across tissues, present in most embryonic and adult cell types [15]. In addition, the protein localizes to multiple subcellular compartments, including the plasma membrane, endosomes, lysosomes, mitochondria, and the nucleus [4,16–19]. However, the phenotypic consequences of *Mgrn1* loss suggest that it functions with remarkable specificity, targeting distinct substrates within specific pathways. Thus, the prevailing question is, how does a single broadly expressed E3 ligase precisely coordinate such varied activities?

Recent studies have expanded the role of MGRN1 in melanoma, linking its expression to the regulation of tumor cell proliferation, motility, and invasive behavior, as well as to patient outcomes [20,21]. In particular, MGRN1 has been proposed as a potential prognostic biomarker for melanoma. Mechanistically, loss of MGRN1 has been associated with increased genomic instability, including elevated DNA damage, as well as alterations in cell adhesion and motility [22]. These studies suggest that MGRN1 may influence melanoma severity by affecting genome maintenance, cell behavior, and immune signaling, thereby extending its functional relevance beyond developmental pathways.

The goal of the present review is to provide a cohesive view of MGRN1 biology by closely examining existing work. To begin, we used the phenotypes of *Mgrn1* mutant mice as an entry point to visualize the many diverse functions of this E3 ligase (Figure 1 and Table 1). We then connected these observations to mechanistic insights from recent signaling, trafficking, and structural studies. By examining these studies together, we aim to highlight unifying themes governing MGRN1’s activity, examine how it targets specific substrates, and outline opportunities for future investigation.

## Abnormal pigmentation and energy homeostasis: MGRN1’s regulation of melanocortin signaling

**Figure 2:**
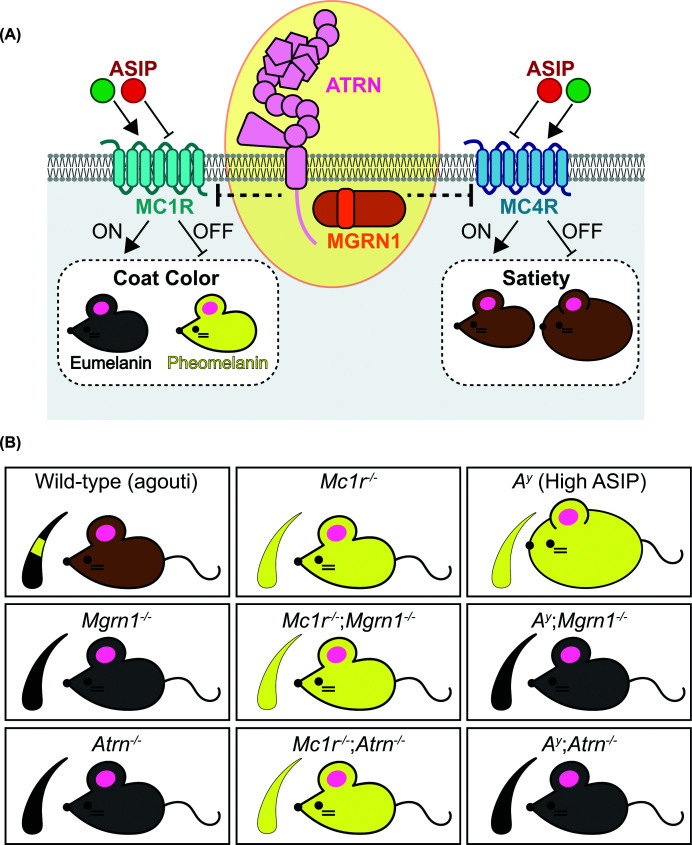
Genetic interactions show that MGRN1 and ATRN inhibit melanocortin signaling facilitated by MC1R and MC4R (**A**) A model depicting the inhibition of MCR function by MGRN1 and ATRN. Melanocortin receptors 1 and 4 (MC1R and MC4R) are GPCRs that regulate coat color and satiety. Their activity is modulated by ligands like ASIP, which acts as an antagonist by directly binding to and suppressing receptor activity. (**B**) A summary of mouse coat color and satiety phenotypes associated with mutations in *Mgrn1, Atrn*, *Mc1r*, and *Asip* [10–12]. MC1R is a key regulator of coat color. When MC1R is active, it stimulates eumelanin (black pigment) production, and when inactive, it leads to pheomelanin (yellow pigment) production. Wild-type (agouti) mice exhibit a banded hair phenotype, where eumelanin (black) is present at the hair base and tip, while pheomelanin (yellow) is present mid-shaft, resulting in a brown coat. Loss-of-function mutations in either *Mgrn1* or *Atrn* result in a darker coat due to a loss of pheomelanin production. In contrast, *Mc1r^−/−^* mutants have a fully yellow coat due to the loss of MC1R-mediated eumelanin production. The additional loss of *Mgrn1* or *Atrn* on an *Mc1r^−/−^* background has no influence on coat color, suggesting that the inhibition by MGRN1 and ATRN is at the same level or upstream of MC1R. Lastly, mice that carry the spontaneous *A^Y^* mutation overexpress ASIP, leading to inhibition of both MC1R and MC4R activity and, consequently, mice that are both yellow and obese. The additional loss of *Mgrn1* and *Atrn* on an *A^Y^* background can reverse these phenotypes, producing non-obese mice with dark coat colors. Collectively, these epistasis studies reinforce the role of MGRN1 and ATRN in suppressing melanocortin signaling via both MC1R and MC4R receptors.

The melanocortin receptors (MCRs) comprise a small subfamily of G-protein-coupled receptors (GPCRs) that regulate a wide range of physiological processes, including pigmentation, adrenal steroidogenesis, immune responses, exocrine secretion, energy balance, and appetite control [23]. Among the five MCRs, MC1R and MC4R show the clearest functional connection to MGRN1. Epistasis experiments in mice demonstrated that loss of *Mgrn1* altered the coat color (MC1R-dependent) and suppressed obesity (MC4R-dependent) in mice carrying the lethal yellow (*A^y^*) mutation, which is associated with ectopic over-expression of the endogenous MCR inverse agonist, agouti signaling protein (ASIP) (Figure 1 and Table 1) [10–12,24]. This placed *Mgrn1* genetically downstream of *Asip*, while crosses to *Mc1r* null mutant mice revealed that the *Mgrn1* coat color phenotype is only observed in mice with a functional MC1R [10]. These results provided one of the earliest pieces of evidence that MGRN1 functions as a negative regulator of melanocortin signaling (Figure 2), but the mechanisms by which it does so remained unresolved. Interestingly, ASIP-MC1R signaling is not limited to canonical cyclic AMP (cAMP) regulation. Studies have shown that while ASIP can suppress cAMP independent of MGRN1 and Attractin (ATRN), its effects on melanocyte behavior and pigmentation still require both MGRN1 and ATRN. This suggests that MGRN1 contributes to a distinct, cAMP-independent component of melanocortin signaling [25].

### Ubiquitin-dependent mechanisms

**Figure 3:**
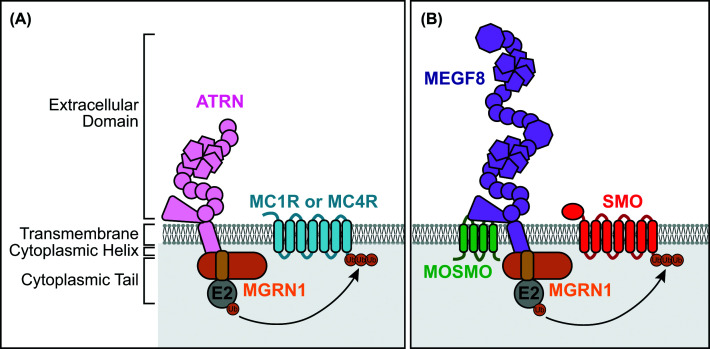
Adapter-mediated recruitment of MGRN1 to distinct cell surface receptors (**A**) In the melanocortin signaling pathway, MGRN1 is recruited to the plasma membrane by the single-pass transmembrane protein ATRN to target the melanocortin receptors MC1R and MC4R. (**B**) In the Hedgehog signaling pathway, MGRN1 is recruited to the plasma membrane through interaction with the transmembrane protein MEGF8. Structural and biochemical studies indicate that MEGF8 positions MGRN1 beneath the membrane, enabling ubiquitination of the GPCR Smoothened (SMO) and promoting its removal from the cell surface. Together, these schematics illustrate a shared adapter-mediated mechanism in which transmembrane proteins spatially constrain MGRN1 to specific signaling receptors, providing a model for understanding how this membrane-tethered E3 ligase achieves substrate specificity across distinct pathways.

Given that MGRN1 is an E3 ubiquitin ligase, one of the earliest proposed models was that it directly ubiquitinates MCRs, promoting their degradation and inhibiting signaling activity. Early experimental support for the present model came from mouse studies demonstrating that MGRN1-dependent regulation of MC1R depends on its ubiquitin ligase activity [26] and from biochemical assays showing that MGRN1 can interact with MC1R, MC2R, and MC4R [19,27] (Table 2). An additional critical clue came from studies on ATRN, a single-pass transmembrane protein whose expression is disrupted in *mahogany* (*mg*) mice [10]. Loss of *Atrn* produces pigmentation and metabolic phenotypes that closely parallel those of *Mgrn1* mutants, suggesting that ATRN and MGRN1 function within a shared pathway to regulate MC1R and MC4R signaling (Figure 2 and Table 3). Consistent with this finding, multiple groups have recently shown that MGRN1 directly interacts with the cytoplasmic tail of ATRN [5,6,8,28]. Importantly, these studies have also shown that MGRN1 alone is not sufficient to ubiquitinate MC1R or MC4R; instead, ATRN is required to enable receptor ubiquitination and degradation [5,19]. Collectively, these findings support a model in which ATRN acts as a transmembrane adapter that recruits MGRN1 to the cell surface, enabling the ubiquitination and turnover of the MCRs [5] (Figure 3A).

**Table 2:** Reported MGRN1 interactions

Protein	Name	MGRN1 interacting domain	Reference
ATRN	Attractin	Pre-engager, engager, RING	[5,28]
ATRNL1	Attractin-like protein 1	Pre-engager, engager, RING	[5]
AMFR	Autocrine motility factor receptor	Pre-engager and engager	[60]
ARRB1	Beta-arrestin-1	Not defined	[30]
ARRB2	Beta-arrestin-2	Not defined	[30]
HSP70	Heat shock protein 70	Not defined	[50]
MC1R	Melanocortin receptor 1	Not defined	[5,19,30]
MC4R	Melanocortin receptor 4	Not defined	[5,19]
MEGF8	Multiple epidermal growth factor-like domains protein 8	Not defined	[4]
NEDD4	E3 ubiquitin-protein ligase NEDD4	Not defined	[26]
PRIO	Major prion protein	Engager	[49]
TSG101	Tumor susceptibility gene 101 protein	Disordered tail (sequence PSAP)	[52]

Protein	Name	CG9941 interacting domain (Drosophila homolog of MGRN1)	Reference
DSD	Distracted	Not defined	[8]

Protein	Name	LOG2 interacting domain (plant homolog of MGRN1)	Reference
GDU1	Glutamine dumper 1	Engager	[7]

**Table 3:** Table of phenotypes in *Atrn^−/−^* mutant mice

Phenotype	Pathway	Source
Abnormal hair pigmentation	Melanocortin signaling	[61]
Elevated basal metabolic rate and suppresses obesity in yellow obese mouse strain (*A^y^*)	Melanocortin signaling	[10,61–64]
Spongiform neurodegeneration	Mitochondrial function	[10]
Hypomyelination	Unknown	[59]

An alternative ubiquitin-dependent model proposes that MGRN1 regulates signaling by facilitating receptor-dependent ubiquitination of β-arrestins rather than directly targeting the MCRs (Table 2). β-arrestins are central mediators of GPCR feedback inhibition, promoting receptor internalization and the removal of G proteins from activated receptors [29]. In this framework, the MCRs serve as scaffolds that mediate MGRN1-dependent ubiquitination of β-arrestins, thereby promoting receptor internalization and desensitization [30].

A third ubiquitin-dependent model proposes that MGRN1 inhibits melanocortin signaling indirectly by ubiquitinating an intermediate component rather than the receptors themselves. A leading candidate for such an intermediate is TSG101, a core component of the Endosomal Sorting Complex Required for Transport (ESCRT-I) that directly binds to and is ubiquitinated by MGRN1 [16,31] (Table 2). The identification of TSG101 as an MGRN1 substrate suggests that MGRN1 may broadly influence receptor sorting and trafficking to lysosomes. However, transgenic mouse studies have clearly shown that disruption of the MGRN1-TSG101 interaction *in vivo* does not impair MC1R regulation [26], indicating that ESCRT-dependent trafficking is not the primary mechanism underlying MCR inhibition. Thus, while MGRN1 clearly engages the ESCRT-I pathway in other contexts (Table 1), MCRs are likely regulated through a different mechanism.

### Ubiquitin-independent mechanisms

In addition to ubiquitin-dependent models, several ubiquitin-independent mechanisms have been proposed to explain how MGRN1 modulates melanocortin signaling. One model suggests that MGRN1 inhibits MCR activity by competing with the G-protein alpha subunit (Gα_s_) for receptor binding [19]. As Gα_s_ activates adenylate cyclase to stimulate cAMP production, such competition would directly suppress receptor output [23,32]. While MGRN1 may play a broader role in regulating GPCRs, the present model does not readily account for the full spectrum of *in vivo* phenotypes observed in *Mgrn1* mutant mice, as previously discussed [33]. Lastly, beyond receptor-level regulation, MGRN1 has also been proposed to influence pigmentation through direct effects on melanosome physiology. Loss of *Mgrn1* in melanocytes increases eumelanin content (Figure 2 and Table 1). The present model proposes that a reduction in *Mgrn1* neutralizes the pH of the melanosome by increasing expression of the melanosome-localized calcium channel Mucolipin 3 (MCOLN3) and other pH-regulatory factors [34]. While this mechanism addresses the pigmentation phenotypes observed in *Mgrn1*^−/−^ melanocytes, it does not explain the defects in energy homeostasis and appetite control, which are mediated by other MCRs.

Collectively, these studies propose multiple models through which MGRN1 regulates melanocortin signaling. A compelling combination of mouse genetics and biochemical assays supports a central role for MGRN1 in regulating MC1R and MC4R. Here, the introduction of the ATRN–MGRN1 complex provides a particularly compelling mechanism for receptor-specific ubiquitination and signaling suppression. At the same time, the additional ubiquitin-dependent and -independent mechanisms highlight the potential functional versatility of MGRN1 across cell types. Through this focused look at melanocortin signaling, we begin to see a unifying theme emerging where MGRN1 achieves signaling specificity through context-dependent substrate recruitment rather than indiscriminate ubiquitination. MGRN1 interacts with many proteins, and its targeting specificity appears to be shared by adapter proteins and scaffolding interactions that spatially constrain its E3 ligase activity (Table 2). This paradigm provides an important foundation for understanding how MGRN1 can engage with many diverse substrates across cell types and subcellular compartments while producing pathway-specific outcomes.

## Congenital malformations: MGRN1’s regulation of Hedgehog and Nodal signaling

Evidence for a fundamental role of MGRN1 in embryonic development first emerged from mouse models, where approximately half of *Mgrn1* loss-of-function mutants died before weaning [13,31]. Closer examination of embryos revealed a spectrum of congenital heart malformations, many of which were likely due to left-right patterning defects (Figure 1 and Table 1). Early studies of the congenital malformations were complicated by incomplete lethality and variable phenotypic severity. This ambiguity was later resolved through the identification of RNF157, a vertebrate-specific paralog of MGRN1. While some *Mgrn1^−/−^* mice survive to adulthood and *Rnf157^−/−^* mice have no overt phenotype, combined loss of *Mgrn1* and *Rnf157* results in fully penetrant embryonic death due to severe cardiac defects [4]. These findings revealed a previously unappreciated functional redundancy between MGRN1 and RNF157 that buffers critical developmental processes.

A major mechanistic advance in understanding the congenital malformations observed in *Mgrn1* mutant mice came from studies focused on the Hedgehog signaling pathway. Genome-wide CRISPR screens identified *Mosmo*, *Megf8*, and *Mgrn1* as novel attenuators of Hedgehog pathway activity [35]. Subsequent biochemical and genetic analyses revealed that these proteins function together as the MOSMO/MEGF8/MGRN1 (MMM) complex. In this complex, the tetraspan MOSMO facilitates the trafficking of the single-pass transmembrane protein MEGF8 to the cell surface, where MEGF8 recruits MGRN1 via interaction with its cytoplasmic tail [4,36]. Once at the plasma membrane, MGRN1 ubiquitinates the GPCR Smoothened (SMO), a critical effector of Hedgehog signaling, thereby promoting SMO removal from the cell surface and suppressing pathway activity (Figure 3B). High-resolution cryo-electron microscopy structures of the MMM complex provided additional insight into this mechanism, revealing how MGRN1 wraps around the cytoplasmic tail of MEGF8 and how MEGF8’s transmembrane helix extends into the cytoplasm and positions MGRN1 at an optimal distance below SMO to facilitate its ubiquitination [5]. By promoting the removal of SMO from the cell surface, the MMM complex functions as a membrane-tethered ubiquitinating module that regulates a cell’s sensitivity to Hedgehog ligands. Consistent with the present model, loss of MMM components results in overlapping developmental phenotypes, including congenital heart malformations, craniofacial abnormalities, left-right patterning defects, and polydactyly, which can be caused by elevated Hedgehog signaling activity [4,13,36,37] (Figure 1 and Tables 1 and 4).

**Table 4:** Table of phenotypes in *Megf8^−/−^* mutant mice

Phenotype	Pathway	Source
Abnormal bone structure	Hedgehog signaling	[4,65]
Abnormal heart development	Hedgehog signaling	[4,65,66]
Abnormal left-right patterning	Nodal signaling	[4,37,65,66]
Abnormal axon guidance in the peripheral nervous system	BMP signaling	[65]
Polydactyly	Hedgehog signaling	[4,65]

Importantly, these Hedgehog-associated defects provide a mechanistic framework for interpreting earlier observations of disrupted left-right patterning in *Mgrn1* mutant embryos. Previous studies have shown that defects in Hedgehog signaling can affect Nodal signaling activity [38,39]. *Mgrn1* mutants have altered expression of Nodal pathway targets, including *Lefty1*, *Lefty2*, and *Pitx2* [13]. In addition, pharmacological inhibition of Hedgehog signaling to suppress the elevated Hedgehog signaling observed in *Mgrn1* mutant embryos was sufficient to partially rescue the cardiac defects [4]. Thus, these data support a model in which the observed disruptions in left-right patterning are a secondary consequence of perturbed Hedgehog signaling.

Collectively, these studies highlight an emerging theme: MGRN1 functions as a membrane-tethered E3 ligase whose ability to specifically target receptors is mediated through interactions with transmembrane adapters (Figure 3). Here we see that in the Hedgehog pathway, MGRN1 binds to MEGF8 to target SMO. This is reminiscent of how MGRN1 binds to ATRN to target MC1R and MC4R in the melanocortin pathway. Notably, ATRN and MEGF8 are paralogs with highly conserved transmembrane and intracellular motifs. Remarkably, a similar membrane-tethered ubiquitination strategy is also observed in plants. In this system, Loss of GDU 2 (LOG2), a plant ubiquitin ligase with high similarity to MGRN1, binds to a single-pass transmembrane protein, glutamine dumper 1 (GDU1), to regulate amino acid transport [7]. These examples support the idea that adapter-mediated recruitment represents a reusable strategy that spatially localizes MGRN1 and allows it to selectively target receptors.

## Spongiform neurodegeneration: MGRN1’s regulation of proteostasis and mitochondrial homeostasis

Another phenotype shared between *Mgrn1^−/−^* and *Atrn^−/−^* mutant mice is spongiform neurodegeneration [14,40,41] (Figure 1 and Tables 1 and 3). This pathology is defined by widespread vacuolation of neural tissues, accompanied by neuronal dysfunction and gliosis, ultimately leading to cognitive decline [42,43]. Although spongiform neurodegeneration is most commonly associated with prion diseases, similar vacuolar pathology has also been observed in Alzheimer’s disease, Lewy body dementia, and certain metabolic disorders, suggesting that this phenotype is a product of multiple cellular stresses [44–47]. Despite the clear and highly penetrant phenotype, the molecular pathways through which loss of *Mgrn1* and *Atrn* drives neurodegeneration remain poorly defined. The differences in age of onset (6–8 weeks in *Atrn* mutants and 10–12 months in *Mgrn1* mutants) [14] suggest that RNF157 can partially compensate for the loss of *Mgrn1*, as observed during embryonic development [4]. Current studies suggest that MGRN1 serves a protective role in the brain through at least two mechanisms: maintaining cellular proteostasis by clearing misfolded proteins and preserving mitochondrial homeostasis.

### Proteostasis and endolysosomal regulation

As an E3 ligase, MGRN1 is well-positioned to regulate protein quality control pathways that rely on ubiquitin-dependent degradation. One model proposes a protective role for MGRN1 in neurons, in which it translocates to the nucleus and promotes an adaptive transcriptional stress response, including up-regulation of the stress-responsive transcription factor ATF3 [48]. ATF3 regulates gene expression programs involved in cellular stress adaptation, and thus, its induction in the context of elevated nuclear MGRN1 suggests a role in supporting neuronal resilience under proteotoxic stress [17]. An alternative model is that MGRN1 protects the brain by promoting the trafficking of misfolded proteins to the lysosome [16,49,50]. MGRN1 directly ubiquitinates the ESCRT-I component TSG101, a key regulator of endosomal sorting and lysosomal degradation [16]. Recent proteomic analyses of neuronal endosomes have identified MGRN1 as an endosome-associated protein, supporting a broader role in endolysosomal trafficking pathways [51]. In line with this, loss of *Mgrn1* delays degradation of epidermal growth factor receptor [16,52,53]. Defects in cargo sorting and receptor turnover contribute to neurodegenerative disease [54]. Notably, spongiform neurodegeneration observed in *Mgrn1* mutant mice occurs in the absence of widespread protein aggregation, a common hallmark of many neurodegenerative disorders. This finding indicates that neurons can be sensitive to defects in trafficking and turnover even in the absence of overt protein aggregation, suggesting a role for MGRN1 in proteostasis that extends beyond classical aggregate clearance mechanisms.

### Mitochondrial homeostasis

A second, and potentially complementary, mechanism by which MGRN1 may protect the brain is its role in preserving mitochondrial homeostasis. Multiple studies indicate that mitochondrial abnormalities precede spongiform neurodegeneration in *Mgrn1* mutant mice [14,18]. Prior to spongiform neurodegeneration, the brains of *Mgrn1*^−/−^ mice exhibit reduced levels of cytochrome c oxidase subunit COX1 (a core component of respiratory complex IV) and a marked increase in protein carbonyls, indicative of oxidative damage [14]. Given the central role of complex IV in the electron transport chain and in regulating reactive oxygen species, these defects suggest early disruption of mitochondrial homeostasis, which can trigger apoptosis and contribute to neurodegenerative disease [55]. Notably, the temporal separation between mitochondrial dysfunction and the appearance of spongiform vacuolation suggests that mitochondrial stress acts as an initiating or sensitizing event rather than the direct cause of neuronal loss. In the present model, impaired mitochondrial homeostasis may lower the threshold for degeneration in aging neurons, rendering them more susceptible to additional cellular stresses.

Collectively, these studies suggest that loss of *Mgrn1* compromises multiple cellular pathways that normally protect neuronal health. Whether disruptions in proteostasis and mitochondrial homeostasis are mechanistically linked or arise independently remains unclear. Currently, the neuronal substrates through which MGRN1 mediates these effects are largely unknown. The shared requirement for both MGRN1 and ATRN in preventing spongiform neurodegeneration suggests that an adapter-mediated, membrane-tethered ubiquitination mechanism (analogous to that observed in the melanocortin and hedgehog signaling pathways) may also operate in this context. In the present scenario, ATRN may localize or constrain MGRN1 to specific neuronal substrates involved in trafficking or mitochondrial maintenance.

## Concluding remarks

MGRN1 is particularly intriguing because it is one of a few known membrane-tethered E3 ubiquitin ligases. Using its unique properties, MGRN1 localizes to the plasma membrane, where it modulates signaling strength by directly regulating receptor abundance. In addition, MGRN1 is important for mitochondrial homeostasis and endosomal trafficking. The high evolutionary conservation of MGRN1 underscores its fundamental biological importance, a conclusion reinforced by the frequently severe phenotypes observed upon *Mgrn1* loss.

The present literature on MGRN1 is broad but loosely connected, leaving major gaps in our understanding. More research is needed to determine how *Rnf157* compensates for the loss of *Mgrn1* and whether this affects the severity of many phenotypes. Additional studies are also needed to extend what is currently known about *Mgrn1’s* role in spongiform neurodegeneration. Although endosomal trafficking and mitochondrial homeostasis are compromised, the mechanisms underlying these phenotypes in the absence of *Mgrn1* remain unclear. Finally, further research is needed to understand how MGRN1 achieves substrate specificity. A clearer picture of this aspect of MGRN1 function could support its use as a therapeutic tool to selectively modulate signaling receptors or to target defined substrates for degradation.

## Perspectives

- MGRN1 represents an understudied class of membrane-tethered E3 ubiquitin ligases that directly regulate signaling receptors, with roles ranging from embryonic development to adult tissue homeostasis.
- Recent biochemical and structural studies have shed new mechanistic light on established genetic models, supporting a model in which MGRN1 achieves substrate specificity through adapter-mediated recruitment by transmembrane proteins, allowing for context-dependent regulation of distinct signaling pathways.
- Identifying additional MGRN1 substrates and clarifying how adapter interactions regulate its activity will be critical to understanding its protective roles in the brain. Improved mechanistic insight into how MGRN1 functions may also allow for new strategies that leverage this E3 ligase to mediate targeted protein degradation.

## Open Access

Open access for this article is provided via our Subscribe to Open programme, made possible through the support of our subscribing institutions. Learn more here: https://portlandpress.com/pages/open_access_options_and_prices#conditional.

## Abbreviations

ASIP: agouti signaling protein
ATRN: Attractin
cAMP: cyclic AMP
Gα_s_: G-protein alpha subunit
GPCRs: G-protein-coupled receptors
GDU1: glutamine dumper 1
LOG2: Loss of GDU 2
MGRN1: Mahogunin Ring Finger 1
md: mahoganoid
mg: mahogany
MCRs: melanocortin receptors
MMM: MOSMO/MEGF8/MGRN1
SMO: Smoothened
